# A quantum algorithm for spin chemistry: a Bayesian exchange coupling parameter calculator with broken-symmetry wave functions†

**DOI:** 10.1039/d0sc04847j

**Published:** 2020-12-24

**Authors:** Kenji Sugisaki, Kazuo Toyota, Kazunobu Sato, Daisuke Shiomi, Takeji Takui

**Affiliations:** Department of Chemistry and Molecular Materials Science, Graduate School of Science, Osaka City University 3-3-138 Sugimoto, Sumiyoshi-ku Osaka 558-8585 Japan sugisaki@sci.osaka-cu.ac.jp sato@sci.osaka-cu.ac.jp takui@sci.osaka-cu.ac.jp; JST PRESTO 4-1-8 Honcho Kawaguchi Saitama 332-0012 Japan; Research Support Department, University Research Administrator Centre, University Administration Division, Osaka City University 3-3-138 Sugimoto, Sumiyoshi-ku Osaka 558-8585 Japan

## Abstract

The Heisenberg exchange coupling parameter *J* (*H* = −2*J****S***_*i*_ · ***S***_*j*_) characterises the isotropic magnetic interaction between unpaired electrons, and it is one of the most important spin Hamiltonian parameters of multi-spin open shell systems. The *J* value is related to the energy difference between high-spin and low-spin states, and thus computing the energies of individual spin states are necessary to obtain the *J* values from quantum chemical calculations. Here, we propose a quantum algorithm, B̲ayesian ex̲change coupling parameter calculator with b̲roken-symmetry wave functions (BxB), which is capable of computing the *J* value directly, without calculating the energies of individual spin states. The BxB algorithm is composed of the quantum simulations of the time evolution of a broken-symmetry wave function under the Hamiltonian with an additional term *j****S***^2^, the wave function overlap estimation with the SWAP test, and Bayesian optimisation of the parameter *j*. Numerical quantum circuit simulations for H_2_ under a covalent bond dissociation, C, O, Si, NH, OH^+^, CH_2_, NF, O_2_, and triple bond dissociated N_2_ molecule revealed that the BxB can compute the *J* value within 1 kcal mol^−1^ of errors with less computational costs than conventional quantum phase estimation-based approaches.

## Abstract 2

A quantum algorithm “Bayesian exchange coupling parameter calculator with broken-symmetry wave function (BxB)” enables us to calculate Heisenberg exchange coupling parameter *J* without inspecting total energies of individual spin states, within 1 kcal mol^−1^ of energy tolerance.

## Graphical abstract

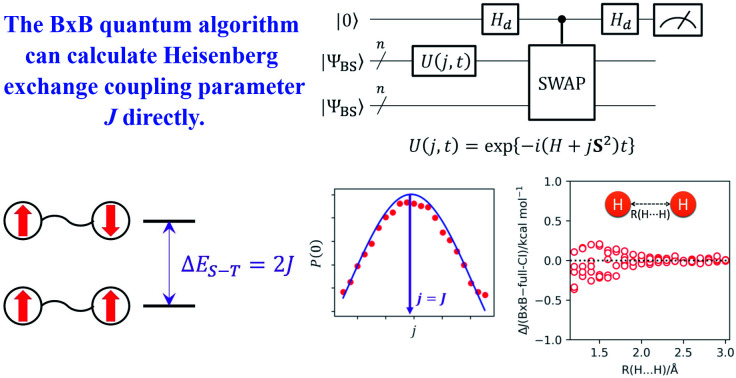

## 1. Introduction

Quantum computing and quantum information processing (QC/QIP) is one of the most innovative research fields in modern science. A quantum computer uses a quantum bit (qubit) as the minimal unit of information, which can possess not only either |0〉 or |1〉 but also arbitrary superposition of the |0〉 and |1〉 states.^1^ By utilising the quantum superposition and entangled quantum states, quantum computers can afford to solve certain problems exponentially faster than the classical counterparts. In 2019, a research team of Google Inc. claimed that they achieved “quantum supremacy”.^2^

Among the diverse topics in the field of QC/QIP, quantum chemical calculations of atoms and molecules are one of the most anticipated applications in the near future. A quantum algorithm based on a quantum phase estimation (QPE) to solve the full configuration interaction (full-CI) in polynomial time, which can give the variationally best wave function within the basis set being used but the computational cost to solve on classical computers increases exponentially against the system size, was reported in 2005.^3^ A quantum–classical hybrid algorithm known as a variational quantum eigensolver (VQE) executable on the noisy intermediate-scale quantum (NISQ) devices^4^ was proposed in 2014.^5,6^ Since then, many reports on the reduction of computational cost with a speedup by improving the quantum algorithms^7–21^ have appeared and relevant experimental demonstrations using various quantum devices^22–30^ have been documented.

Despite of the rapid progress of the theory for quantum chemical calculations on quantum computers (QCC-on-QCs), a method to efficiently treat open shell electronic structures is still in the stage of infancy. Open shell systems are ubiquitous in chemistry. For example, organic biradicals can be used as prototypes of molecular spin quantum computer,^31,32^ polarising agents in dynamic nuclear polarisation (DNP),^32,33^ organic light-emitting materials,^34,35^ and so on. Open shell multi-nuclear transition metal complexes are often involved in enzymes as reactive centres.^36,37^ Single molecule magnets have been extensively studied as molecular memory devices.^38^ To disclose their electronic structures, sophisticated *ab initio* quantum chemical calculations are powerful and essential tools. However, in open shell systems carrying spin-β unpaired electrons, the wave function has a strong multi-configurational character and the Hartree–Fock wave function is not a good approximation of the ground state. We have developed theoretical methods to construct spin symmetry-adapted wave functions,^39–41^ to determine spin quantum numbers of arbitrary wave functions,^42^ and to purify the spin contaminated wave functions,^43^ on quantum computers.

In molecules carrying two or more unpaired electrons, the determination of the ground state spin multiplicity and estimation of the spin state energy gap are important tasks. Experimentally, the spin state energy gap is evaluated *e.g.*, by simulating the temperature dependence of magnetic susceptibility or ESR forbidden transition intensity,^44–46^ by assuming a Heisenberg spin Hamiltonian^47–51^ given in eqn (1).1
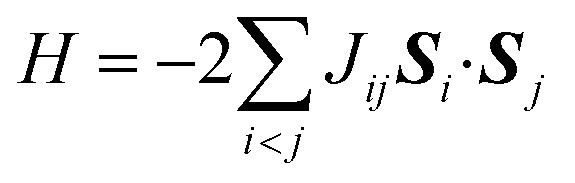
Here, *i* and *j* run over unpaired electrons, *J*_*ij*_ is an exchange coupling parameter, and ***S***_*i*_ is an electron spin operator acting on the *i*-th unpaired electron. Under this definition two spins prefer being ferromagnetic when *J*_*ij*_ is positive. To obtain the exchange coupling parameter *J* from quantum chemical calculations, one must calculate the energies of high-spin and low-spin states and subtract to obtain the energy difference. The situation has been the same even if we use a quantum computer. However, as we propose in this paper, a quantum algorithm “B̲ayesian ex̲change coupling parameter calculator with b̲roken-symmetry wave functions (BxB)” is capable of calculating the *J* value directly, without evaluating the energies of individual spin states. The direct calculation of spin state energy gaps and an associated exchange coupling parameter *J* is very important in QCC-on-QCs, because (1) the *J* value is usually in the order of kcal mol^−1^ or even smaller and therefore chemical precision is necessary to evaluate the value quantitatively, and (2) determining the energy down to the fine order of magnitude on quantum computer is extremely cost demanding. Note that quantum algorithms for the direct estimation of the energy gap by combining Ramsey-type measurement and Rabi oscillation experiments or quantum annealing were proposed recently.^52,53^

The key technique used in the BxB quantum algorithm consists in (1) quantum simulations of the time evolution of a broken-symmetry wave function under the Hamiltonian with an additional term *j****S***^2^, (2) a SWAP test for the estimation of the wave function overlap, and (3) Bayesian optimisation of the *j* parameter. Importantly, the proposed approach is easier to be implemented in the process of quantum computing, compared with conventional QPE-based full-CI, and it can afford to calculate the *J* value within 1 kcal mol^−1^ of errors regardless of the magnitude and sign of the exchange coupling parameter *J*.

This paper is organised as follows. In Section 2, we briefly review the theoretical methods for the calculation of exchange coupling parameter *J* on classical computers, and quantum chemical calculations on quantum computers. Then, we introduce a new quantum algorithm for the *J* value calculations. In Section 3, numerical simulation results of the *J* value calculations for H_2_ molecule under a covalent bond dissociation, singlet–triplet energy gaps of C, O, Si, NH, OH^+^, CH_2_, NF and O_2_ are given. N_2_ molecule under triple bond dissociation is also discussed as a representative example of systems with more than two unpaired electrons. Conclusions and future perspectives are given in Section 4.

## 2. Theory

### 2.1 Quantum chemical calculations of the exchange coupling parameter *J* on classical computers

Let us assume a biradical molecule as a chemical entity which has two unpaired electrons. The electronic ground state is either a spin-singlet (*S* = 0) or spin-triplet (*S* = 1), depending on the nature of magnetic interaction between unpaired electrons. Here, *S* is a spin quantum number. The strength of the magnetic interaction between unpaired electrons is characterised by an exchange coupling parameter *J*, by assuming the Heisenberg spin Hamiltonian given in eqn (1). The eigenvalue of the Heisenberg spin Hamiltonian is 3*J*/2 and −*J*/2 for the spin-singlet and triplet states, respectively.

The quantum chemical calculation is a powerful tool to disclose the electronic structures of open shell molecules and to understand their magnetic interactions. In quantum chemical calculations, the *J* value in two-spin systems can be computed from the singlet–triplet energy gap Δ*E*_S–T_, by using the relationship given in eqn (2).2
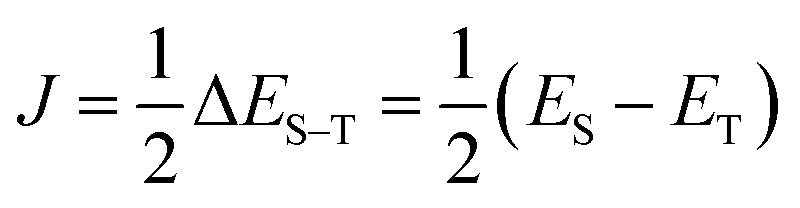


It should be emphasised that the spin-singlet state considered here has two unpaired electrons and thus open shell. If the lowest singlet state is closed shell, the coupled cluster method like CCSD(T) can give reliable Δ*E*_S–T_,^54^ but in the case of the open shell singlet, the Hartree–Fock wave function is not a good approximation and the conventional coupled cluster method is unsuitable. The two-spin open shell singlet wave function is mainly described by the linear combination of two Slater determinants:3
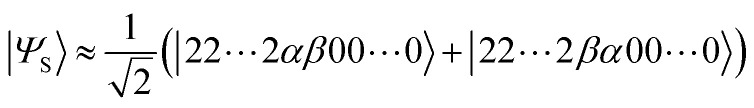
Here, 2, *α*, *β*, and 0 specify doubly occupied, singly occupied by a spin-α electron, singly occupied by a spin-β electron and unoccupied orbitals, respectively. The multi-configurational nature of the open shell singlet wave function originates from the symmetry requirement of the spin operator ***S***^2^.^55,56^ The wave function is a simultaneous eigenfunction of the electronic Hamiltonian *H* and the ***S***^2^ operator, but single Slater determinant carrying spin-β unpaired electrons is not an eigenfunction of the ***S***^2^ operator. The configuration interaction (CI) methods or the multi-configurational methodologies such as complete active space self-consistent field (CASSCF) are required to treat open shell singlet wave functions explicitly. In fact, the multi-reference (MR)-CI, MR-CC, and multi-reference perturbation theory (MRPT) with the CASSCF reference wave function are capable of predicting the Δ*E*_S–T_ and the *J* value in a quantitative manner.^57–59^ In this framework, the energies of high-spin and low-spin states are calculated separately and subtract to obtain the Δ*E*_S–T_ and *J* value.

The fact that the open shell singlet wave function has a multi-configurational character prevents us from directly treating open shell singlet states by means of single configuration theory such as Hartree–Fock (HF) and density functional theory (DFT). Instead, the *J* value can be calculated by using Yamaguchi's equation given in eqn (4) in conjunction with the broken-symmetry (BS) wave function |*Ψ*_BS_〉.^60,61^ The |*Ψ*_BS_〉 is a spin-mixed wave function, and in two-spin systems it is a linear combination of the spin-triplet wave function with *M*_s_ = 0 and open shell singlet wave function as given in eqn (5).4
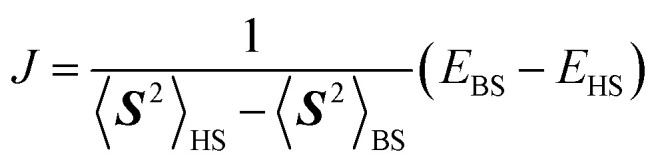
5
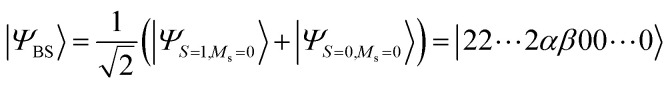
Here, 〈***S***^2^〉 is an expectation value of the ***S***^2^ operator. For two-spin systems HS corresponds to the spin-triplet state. The *E*_HS_ can be easily calculated at the single configuration theory because the spin-triplet wave function with *M*_s_ = 1 is generally well approximated by the single Slater determinant |22⋯2*αα*00⋯0〉. Thus, the energy calculations of the high-spin and BS states are required to obtain the *J* value from Yamaguchi's equation.

Apart from the methods above, Δ*E*_S–T_ can be calculated from the spin-flip variants of the time-dependent DFT, CI, and equation-of-motion coupled cluster (EOM-CC) methods.^62–65^ In these methods the high-spin state is calculated by DFT, HF, and CC, respectively, and the low-spin state is described by the spin flip excitations from the high-spin reference state. In these approaches, Δ*E*_S–T_ is calculated as an excitation energy from the high-spin state.

As described here, the calculations of Δ*E*_S–T_ and the *J* value generally require two separate calculations, *i.e.* the energy calculations of the high-spin and low-spin states.

### 2.2 Quantum chemical calculations on quantum computers

Here we briefly review the two major theoretical methods for quantum chemical calculations on quantum computers, namely QPE-based full-CI and VQE. We also discuss the conventional approach to calculate the *J* value on a quantum computer.

**Fig. 1:**
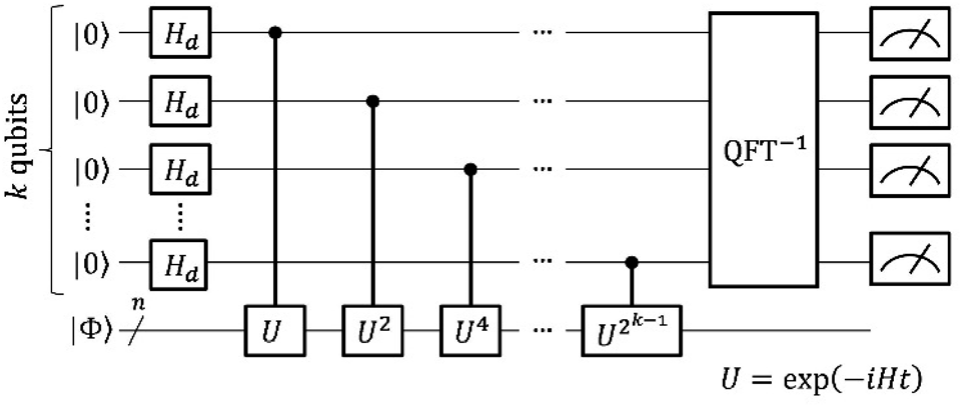
A quantum circuit of the quantum phase estimation-based full-CI calculations.

A quantum circuit for QPE-based full-CI is given in Fig. 1. In Fig. 1, the horizontal lines specify a qubit or *n*-qubit quantum register, and squares, circles and vertical lines represent quantum gates. Detailed definitions of the quantum gates and quantum circuits are given in the ESI.†

In QPE the time evolution of a wave function |*Φ*〉 is simulated conditional on the qubits for readout and the relative phase shift caused by the time evolution is extracted by means of inverse quantum Fourier transformation (QFT^−1^) and following qubit measurements.^66^ The quantum state |*Φ*〉 is projected onto the eigenstate of Hamiltonian by the measurements and thus the full-CI energy can be obtained. Which electronic state is obtained in QPE is probabilistic, depending on the square overlap |〈*Φ*|*Ψ*_*i*_〉|^2^. Here, |*Ψ*_*i*_〉 is the full-CI wave function of the *i*-th electronic state. By preparing the |*Φ*〉 to have sufficiently large overlap with the targeted electronic state, the QPE is capable of giving the full-CI energy of a desired electronic state with high probability. In closed shell singlet molecules around their equilibrium geometry or high-spin molecules having no spin-β unpaired electrons, the Hartree–Fock wave function |*Ψ*_HF_〉 is generally a good approximation of the ground state. In open shell molecules carrying spin-β unpaired electrons, one can use a configuration state function |*Ψ*_CSF_〉 that is an eigenfunction of the ***S***^2^ operator as |*Φ*〉. The |*Ψ*_CSF_〉 can have a large overlap with the full-CI wave function, and it can be easily prepared on quantum computers.^39,40^

In QCC-on-QCs the second-quantised Hamiltonian in eqn (6) is transformed into a qubit Hamiltonian by using Jordan–Wigner transformation (JWT),^3,67^ Bravyi–Kitaev transformation (BKT),^68,69^ or other methods,^3,68,70^ and the wave function is mapped onto a quantum register. In this paper, we used the JWT for wave function mapping. In the JWT, the fermionic creation and annihilation operators *a*^†^_*p*_ and *a*_*q*_ are transformed to the tensor products of Pauli operators (Pauli strings) as given in eqn (7) and (8), respectively, and the qubit Hamiltonian is expressed by the linear combination of Pauli strings as in eqn (9) and (10).6
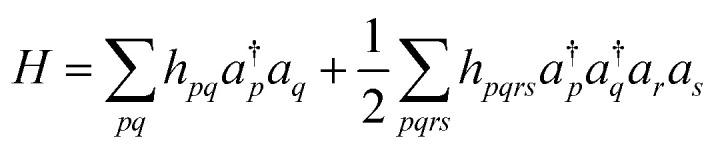
7
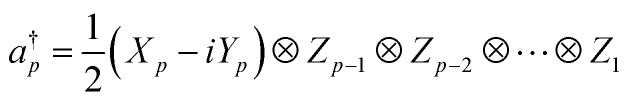
8
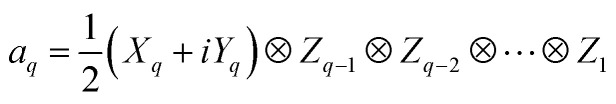
9
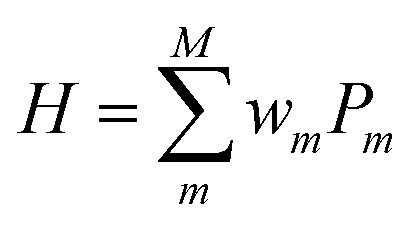
10
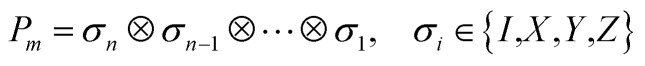
Here, *h*_*pq*_ and *h*_*pqrs*_ in eqn (6) denote one- and two-electron integrals, respectively. *X*_*i*_, *Y*_*i*_, and *Z*_*i*_ are Pauli operators acting on the *i*-th qubit. Under the JWT, each qubit represents the occupation number of a particular spin orbital, and the number of qubits required for the wave function mapping is equivalent to the number of spin orbitals included in the calculations.

The qubit Hamiltonian contains many noncommutative Pauli strings and Trotter decomposition is often adopted to construct the quantum circuit corresponding to the time evolution operator *U* = exp(−*iHt*). The time evolution operator under the first and second order Trotter decompositions are given in eqn (11) and (12), respectively.11
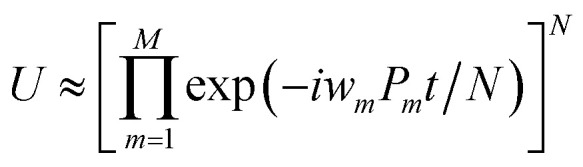
12
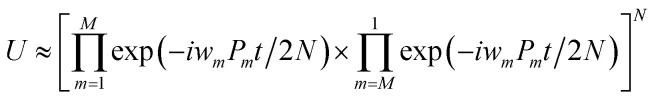


The quantum circuit for the time evolution operator exp(−*iwX*_1_*Z*_2_*Z*_3_*X*_4_*t*) is illustrated in Fig. S1 in ESI† as an example.

The QPE-based full-CI method is a powerful tool in QCC-on-QCs, but there are several challenging issues for the implementation on real quantum devices. First, the quantum circuit is so deep that only small molecules with minimal basis sets can be currently calculated within decoherence time of qubits. Second, the quantum circuit contains many controlled-*R*_*z*_ gates and they should be executed with high fidelity to obtain the reliable energy. Third, the evolution time *t* becomes very long when we try to determine the energy to a small order of magnitudes. The *k*-qubit QPE described in Fig. 1 can compute the full-CI energy with an energy tolerance Δ*E* = π/2^*k*−1^*t* for *U* = exp(−*iHt*). If we set *t* = 1 we need *k* = 12 to achieve ∼1 kcal mol^−1^ of energy tolerance. In this case, we have to apply the time evolution operator up to *U*^2^11^^ = exp(−2^11^*iH*). From these reasons it is believed that quantum error correction code is necessary for the implementation of QPE.

In contrast to QPE, VQE is computationally less demanding, and it is expected to be executable on NISQ devices available in the near future. In VQE, the quantum processing unit repeatedly performs the wave function preparation using the parametrised quantum circuit and following measurements to calculate the energy expectation value in eqn (13). The classical processing unit performs a variational optimisation of the parameter *θ* and feedbacks the parameter to the quantum processing unit.

13 *E*(*θ*) = 〈*Φ*_0_|*U*^†^(*θ*)*HU*(*θ*)|*Φ*_0_〉

In eqn (13), the |*Ψ*_HF_〉 is generally used as |*Φ*_0_〉. *U*(*θ*) determines the quality of wave function. So far, unitary coupled cluster (UCC)^5,6,71^ and heuristic ansatzes^25,26,72^ have been mainly used to construct *U*(*θ*). In VQE, the energy expectation value *E*(*θ*) is calculated through repeated measurements and therefore it includes statistical errors. It is known that approximately *O*(*w*_max_^2^/*ε*^2^) of measurements are required to determine the energy within a precision *ε*, where *w*_max_ = max(|*w*_*m*_|) in eqn (9).^26^ Millions of measurements are required to calculate the energy within 1 kcal mol^−1^ of tolerance in VQE for molecules containing second-row atoms or heavier.

Noteworthily, to calculate the Heisenberg exchange coupling parameter *J* by using QPE or VQE, two separate energy calculations for the high-spin and low-spin states are necessary, just as on classical computers. Here, we emphasise that the calculation of the *J* value precisely is much more difficult on quantum computers than on classical computers, because the *J* value is generally in the order of kcal mol^−1^ or even smaller. To discuss the spin state energy gap and *J* value within 1 kcal mol^−1^ of tolerance, we have to calculate the energy of individual spin states within 0.5 kcal mol^−1^ of precision. In both QPE and VQE, the computational cost steeply depends on the requested energy precisions; the length of the time evolution must be doubled in QPE, and the measurement number should be quadrupled in VQE, to make the energy precision *ε* to be half.

We emphasise that most of chemistry problems including the *J* value calculation focus on the energy difference or relative energy, rather than the total energy itself. From the viewpoint of computational cost reduction and accuracy improvement, it is very useful if we can calculate the energy difference between two electronic states directly in one calculation, without inspecting the total energy of individual electronic states. In the next section, we show that the direct calculation of the *J* value is possible by using the BxB quantum algorithm, if the system can be approximated by the two-site Heisenberg spin Hamiltonian.

### 2.3 A BxB quantum algorithm for the calculation of Heisenberg exchange coupling parameter *J*

As discussed above, the calculation of the spin state energy gap and Heisenberg exchange coupling parameter *J* generally requires two separate calculations to evaluate the total energies of high-spin and low-spin states, regardless of whether calculations are performed on classical or quantum computers. However, because quantum computers utilise quantum superposition states as the computational resource, we can calculate the spin state energy gap and *J* value directly on quantum computers.

Unless otherwise specified we focus on two-spin systems. The theory can be easily extended to the systems carrying more than two unpaired electrons if the system is approximated by the two-site Heisenberg model 
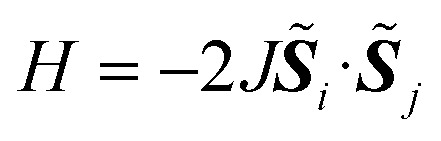
 with effective spins *S̃* ≥ 1/2.

According to the discussions in Section 2.1, the ground state of two-spin systems is either spin-singlet (*J* < 0) or spin-triplet (*J* > 0) state. The energy difference between the lowest spin-singlet and triplet states Δ*E*_S–T_ equals to 2*J*, as given in eqn (2). Importantly, both the spin-singlet and triplet wave functions are simultaneous eigenfunctions of the Hamiltonian and the spin operator ***S***^2^. The eigenvalue of the ***S***^2^ operator is *S*(*S* + 1), where *S* is a spin quantum number. By using this feature, we can reformulate the problem to calculate the *J* value to another form.

Let us focus on the shifted Hamiltonian *H*′ with an additional term *j****S***^2^ as in eqn (14).

14 *H*′ = *H* + *j****S***^2^

*H*′ and *H* have the simultaneous eigenfunctions, because [*H*, ***S***^2^] = 0. The eigenvalue of the shifted Hamiltonian *H*′ is given in eqn (15).

15 *E*′ = *E* + *jS*(*S* + 1)

Eqn (15) stands for 
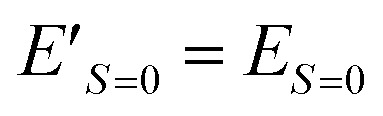
 and 
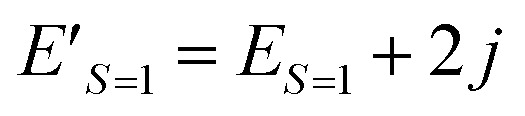
. From this relationship we can derive the following important theorem:

#### Theorem 1

The problem to calculate the energy gap between two states belonging to different spin quantum numbers is equivalent to the problem to find the *j* parameter in the shifted Hamiltonian *H*′ = *H* + *j****S***^2^, where the two spin states have the same eigenvalue of *H*′. The energy gap is calculated as Δ*E*_LS–HS_ = *j*[*S*_HS_(*S*_HS_ + 1) − *S*_LS_(*S*_LS_ + 1)].

**Fig. 2:**
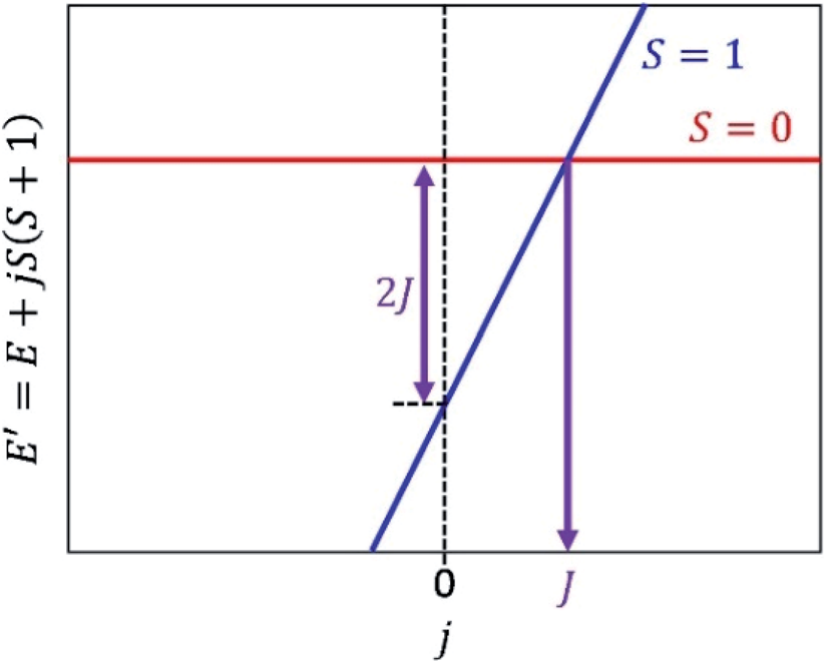
An energy diagram of the shifted Hamiltonian *H*′ = *H* + *j****S***^2^ with *j* as a variable. *J* > 0 assumed.

Fig. 2 illustrates a schematic energy diagram of the shifted Hamiltonian *H*′ with *J* > 0. In the case of *J* < 0, the crossing point of the *S* = 0 and *S* = 1 lines appears on the left hand side of the figure. Importantly, theorem 1 holds not only for two-spin systems but also systems carrying more than two unpaired electrons. Furthermore, the following theorem can also be derived.

#### Theorem 2

If the system can be approximated by the two-site Heisenberg spin Hamiltonian with effective spins *S̃* ≥ 1/2 as given in eqn (16), all spin states have the same eigenvalue of *H*′ if *j* = *J*.16
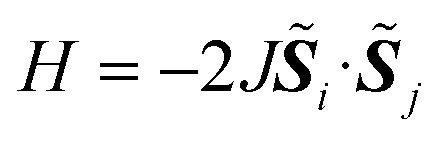


Theorem 2 is derived from the fact that the spin state energy gap in Heisenberg spin Hamiltonian of eqn (16) is in eqn (17). In Section 3, we treat the triple bond dissociation of a N_2_ molecule (*S̃* = 3/2), which involves six unpaired electrons.

17 Δ*E*_*p–q*_ = *E*_*S*=*p*_ − *E*_*S*=*q*_ = *J*[*q*(*q* + 1) − *p*(*p* + 1)]

From theorem 1 the high-spin and low-spin states have the same eigenenergy of the shifted Hamiltonian *H*′ if *j* = *J*. Thus, the calculation of the *J* value can be accomplished by finding the *j* parameter so as for the broken-symmetry wave function |*Ψ*_BS_〉 in eqn (18) to become an eigenfunction of *H*′.

18 |*Ψ*_BS_〉 = *c*_*S*=0_|*Ψ*_*S*=0_〉 + *c*_*S*=1_|*Ψ*_*S*=1_〉

If |*Ψ*_BS_〉 is an eigenfunction of *H*′, the time evolution of |*Ψ*_BS_〉 under the shifted Hamiltonian *H*′ merely gives rise to a phase shift without changing the structure of |*Ψ*_BS_〉. The deviation of |*Ψ*_BS_〉 from the eigenfunction of *H*′ can be estimated from the square overlap |〈*Ψ*_BS_|*U*(*j*, *t*)|*Ψ*_BS_〉|^2^, where *U*(*j*, *t*) is a time-evolution operator defined in eqn (19). The square overlap |〈*Ψ*_BS_|*U*(*j*, *t*)|*Ψ*_BS_〉|^2^ can be efficiently evaluated on a quantum computer, by utilising a SWAP test.^73^

19 *U*(*j*, *t*) = exp(−*iH*′*t*) = exp{−*i*(*H* + *j****S***^2^)*t*}

**Fig. 3:**
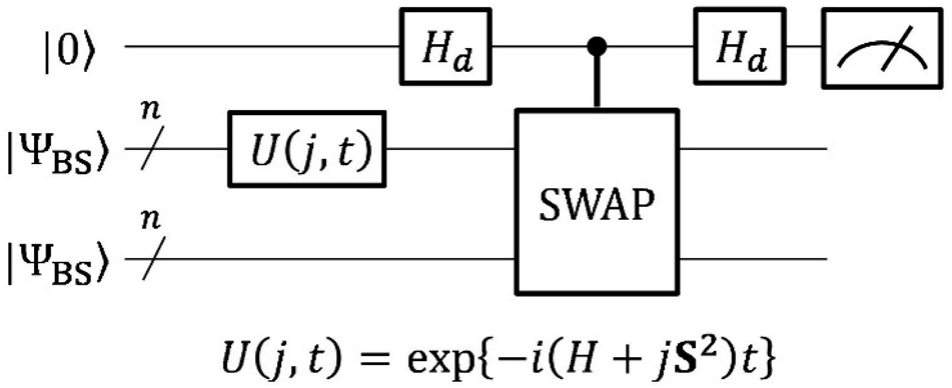
A quantum circuit to estimate the square overlap |〈*Ψ*_BS_|*U*(*j*, *t*)|*Ψ*_BS_〉|^2^ by utilising a SWAP test.

The quantum circuit to estimate the square overlap |〈*Ψ*_BS_|*U*(*j*, *t*)|*Ψ*_BS_〉|^2^ on a quantum computer is depicted in Fig. 3.

The quantum circuit in Fig. 3 consists of two parts: the time evolution of |*Ψ*_BS_〉 under the shifted Hamiltonian *H*′ and a SWAP test. Here, *n* is equal to the number of active spin orbitals in our implementation. The controlled-SWAP gate appearing as the third quantum gate from left conditionally interchanges the quantum states of qubits storing |*Ψ*_BS_〉 and *U*(*j*, *t*)|*Ψ*_BS_〉 if the control qubit is in the |1〉 state. The quantum state before measurement is given in eqn (20).20
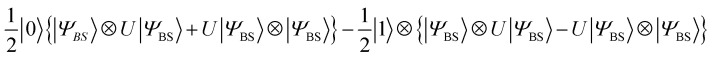


Assuming eqn (5) (
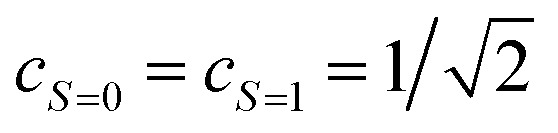
 in eqn (18)), the probability to obtain the |0〉 state *P*(0) is calculated as in eqn (21).21
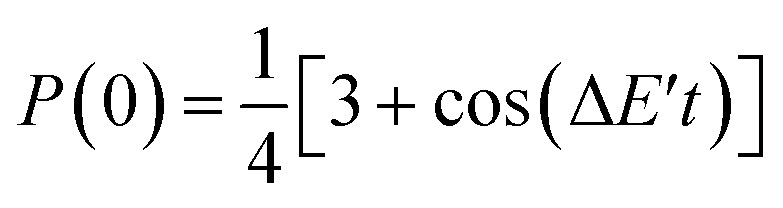
Here, Δ*E*′ denotes an energy difference between the low-spin and high-spin states under the shifted Hamiltonian *H*′.

22 Δ*E*′ = *E*_*S*=0_ − (*E*_*S*=1_ + 2*j*)

For more general situations in which the broken-symmetry wave function is described by a linear combination of many electronic states, the probability *P*(0) is given by eqn (23), where *c*_*i*_ is the expansion coefficient of the *i*-th electronic state in the broken-symmetry wave function.23
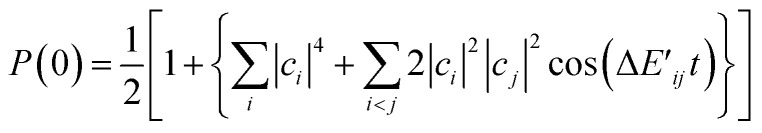


Eqn (21)–(23) reveal that *P*(0) oscillates depending on *j* and *t*, and it gives the maximum value when *j* = *J*. Thus, we can calculate the *J* value by seeking the *j* parameter giving the maximum *P*(0), which can be efficiently done by Bayesian inference, as described below. Note that even though the dynamical electron correlation effect is prominent and contributions from electronic states other than high-spin and low-spin states under study to the broken-symmetry wave function are not negligible, the *j* value giving the maximum *P*(0) does not change much, as long as the corresponding expansion coefficient *c*_*i*_ is small.

In the field of quantum computing, Bayesian inference has been used in QPE^74,75^ and Hamiltonian learning.^76–78^ The Bayesian phase estimation (BPE) is robust to experimental imperfections, noise, and decoherence, and it outperforms iterative QPE algorithms. Quantum Hamiltonian learning is a quantum algorithm to efficiently infer the Hamiltonian of an untrusted quantum simulator using a trusted one.

In the BxB quantum algorithm, we infer the *j* parameter of *U*(*j*, *t*) giving the maximum *P*(0) in the quantum circuit depicted in Fig. 3. A posterior distribution *P*(*j*|0; *t*) is calculated as eqn (24) from Bayes' theorem.24
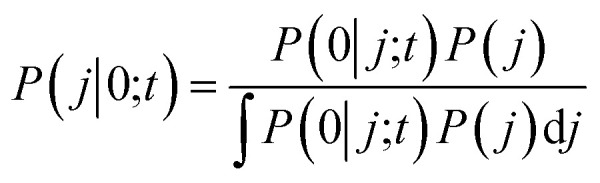
Here, *P*(0|*j*; *t*) is a likelihood function given in eqn (23). *P*(*j*) denotes a prior distribution of *j*. We can calculate the likelihood function *P*(0|*j*; *t*) by repeatedly executing the quantum circuit in Fig. 3 with various *j* values.

The BxB algorithm is executed in the following steps. (1) Gives the prior distribution *P*(*j*). In this work, we used a normal distribution with *μ* = 0 and *σ*^2^ = 1 as the starting prior distribution, where *μ* and *σ*^2^ denote the mean and variance, respectively. (2) The evolution time *t* of *U*(*j*, *t*) is set to be 1.2/*σ*^2^ for two-spin systems. For triple bond dissociation of N_2_ molecule we used *t* = 0.4/*σ*^2^. These evolution time lengths were set so that *P*(0|*j*; *t*) becomes a single maximum when *j* is taken in the range of *μ* − *σ*^2^ to *μ* + *σ*^2^. (3) Draws *m* samples in the range of *μ* − *σ*^2^ to *μ* + *σ*^2^ with a constant interval, and executes the quantum circuit of Fig. 3*R* times with a given *t* and *j* to calculate *P*(0|*j*; *t*). In this work, we used *m* = 21 and *R* = 1000. (4) The likelihood function *P*(0|*j*; *t*) is fitted by a normal distribution and the posterior distribution *P*(*j*|0; *t*) was calculated by using eqn (24). (5) If *μ*(posterior) < *μ*(prior) − *σ*^2^/2 or *μ*(posterior) > *μ*(prior) + *σ*^2^/2, return to step (3) with the *j* value giving the largest *P*(0) as *μ*(prior). Note that this and next steps are introduced for the purpose of algorithm stability enhance. (6) Set *σ*^2^(posterior) = *σ*^2^(prior)/5 if *σ*^2^(posterior) is smaller than *σ*^2^(prior)/5. (7) Convergence check. If *σ*^2^(posterior) is smaller than the threshold, the algorithm returns *μ*(posterior) as the estimate of the *j* value, and otherwise returns to step (2) with the calculated posterior distribution as the prior distribution of the next iteration. In this study, the convergence threshold is set to be 0.001 Hartree. It should be noted that the convergence threshold controls the precision of the calculated *J* value. In case the *J* value of the molecule under study is small (*e.g.*, in the order of cal mol^−1^), tighter threshold should be adopted.

## 3. Results and discussion

In order to demonstrate the BxB quantum algorithm we developed a numerical quantum circuit simulation program using Python with OpenFermion^79^ and Cirq^80^ libraries and performed the numerical simulations for covalent bond dissociation in H_2_, triplet ground states of C, O, Si, NH, OH^+^, CH_2_, NF, and O_2_, and triple bond dissociation in N_2_. The simulations were performed five times for each geometry. The computational conditions for the numerical simulations are summarised in the ESI.†

### 3.1 Covalent bond cleavage in H_2_ molecule

**Fig. 4:**
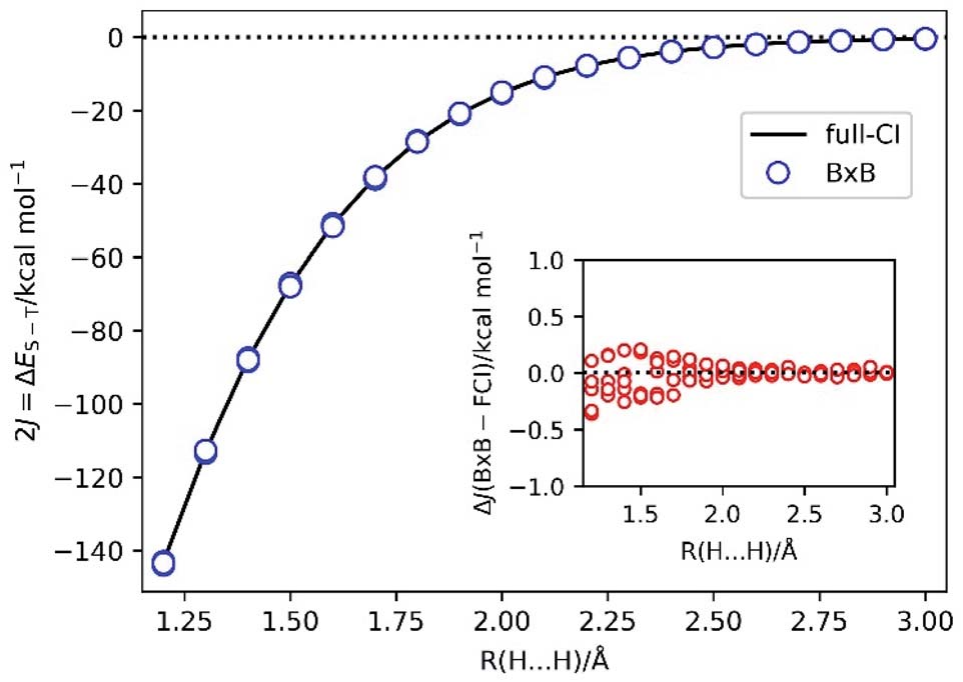
The 2*J* = Δ*E*_S–T_ values of H_2_ molecule under the covalent bond cleavage calculated from the full-CI/STO-3G level (solid line) and from the quantum circuit simulations of the BxB algorithm (blue circles). Inset: the difference of the *J* values obtained from the quantum circuit simulations and full-CI/STO-3G.

To demonstrate the BxB quantum algorithm, we performed the numerical quantum circuit simulations for covalent bond dissociation in H_2_ molecule in the range of 1.2 Å to 3.0 Å. Note that |*Ψ*_BS_〉 is converged to the RHF root for *R*(H–H) = 1.1 Å and shorter. We used the STO-3G basis set and |*Ψ*_BS_〉 is mapped onto four qubits. The 2*J* = Δ*E*_S–T_ value calculated from the full-CI/STO-3G level and that obtained from the BxB quantum algorithm simulations, and difference of the *J* values from the simulations and full-CI are plotted in Fig. 4.

Fig. 4 clearly shows that the BxB algorithm enables us to very accurately calculate the *J* value, and noticeably the deviations from the *J*(full-CI) is less than 0.5 kcal mol^−1^ at all atom–atom distances. The Δ*J* value tends to become large for the shorter H–H distance, which presumably originates from the fact that |*Ψ*_BS_〉 is not well described by the linear combination of the lowest singlet and triplet states, and any contributions from other electronic states become non-negligible. As a result, the likelihood function becomes flatten at shorter bond lengths. Also, for longer bond distances the high-spin and low-spin wave functions are expanded by the same sets of Slater determinants. In this case, Trotter decomposition errors on the high-spin and low-spin states are quite similar and they can be cancelled out when we calculate the energy difference. For shorter bond distances such Trotter error cancellations are not sufficient. Thus, the Δ*J* value can be improved by adopting tighter threshold for convergence check and the larger number of Trotter decomposition slices. In fact, by changing the convergence threshold to 0.0001 Hartree and setting the time for single Trotter step to 0.2 atomic unit, we obtained *J*(BxB) = −33.752 ± 0.001 kcal mol^−1^ at *R*(H–H) = 1.5 Å, which corresponds to Δ*J* = −0.003 kcal mol^−1^.

It should be also emphasised that Bayesian optimisation converged very fast: only 5–8 iterations are required to achieve the convergence (see Fig. S2 in the ESI†). The evolution time *t* in the final Bayesian iteration is about 250–300 atomic unit, which should be compared with *t* ∼ 8000 a.u. to calculate the Δ*E*_S–T_ within the same precision (0.5 kcal mol^−1^ for the energy difference and hence 0.25 kcal mol^−1^ for the total energy) by using the conventional QPE. Although the BxB algorithm requires the repeated execution of the quantum circuit to evaluate the likelihood function, the computational cost reduction owing to the shortened evolution time is remarkable.

### 3.2 Atoms and small molecules with spin-triplet ground states

Next, we studied the ground state spin-triplet atoms and small molecules. The target systems are C, O, Si, NH, OH^+^, CH_2_, NF, and O_2_. The experimental Δ*E*_S–T_ value is available for these systems.^81,82^ Note that |*Ψ*_BS_〉 is approximated by the linear combination of the lowest spin-triplet and the second lowest spin-singlet wave functions in CH_2_ and O_2_, rather than the lowest spin-singlet wave function. Therefore, the *J* value obtained from the BxB algorithm does not correspond to the half of Δ*E*_S–T_ in CH_2_ and O_2_. To calculate the Δ*E*_S–T_ that can be directly compared to the experimental value in CH_2_ and O_2_ molecules, the initial wave function should be replaced from the broken-symmetry wave function to |*Ψ*_0_〉 as defined in eqn (25) and (26), respectively. Here, 2, *α*, and 0 stand for doubly occupied, singly occupied by a spin-α electron, and unoccupied orbitals, respectively.25
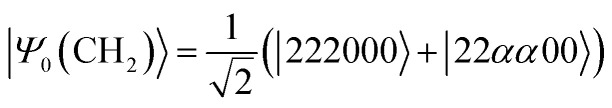
26
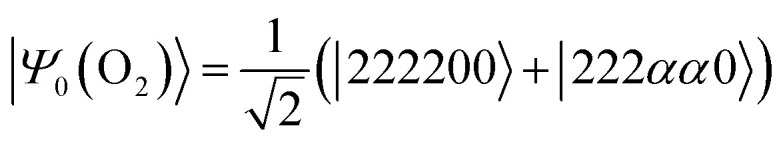


The numerical quantum circuit simulations and quantum chemical calculations were carried out using an active space approximation. For neutral atoms (C, O, and Si) and NH, OH^+^, and CH_2_ all valence orbitals are included in the active space. In NF and O_2_, the valence σ/σ* and π/π* orbitals are considered to be in the active space. The number of qubits required for the wave function mapping is summarised in Table 1.

**Table 1:** Theoretical and experimental *J* values of triplet ground state atoms and small molecules

Systems	Number of qubits^a^	*J*(STO-3G)/kcal mol^−1^		*J*(6-311++G**)/kcal mol^−1^		*J*(Exptl.)/kcal mol^−1^
		CAS-CI	BxB	CAS-CI	BxB	
C	8	22.76	22.59(1)	18.28	18.14(1)	14.57^b^
O	8	29.77	29.60(2)	26.07	25.92(2)	22.68^b^
Si	8	15.57	15.43(1)	12.50	12.41(1)	9.00^b^
NH	10	27.96	27.54(2)	24.80	24.16(2)	19.5^c^
OH^+^	10	31.68	31.17(1)	31.27	30.49(2)	25.25^c^
CH_2_	12	32.37	31.96(4)	24.97	24.64(3)	
NF	12	22.69	22.57(3)	20.64	20.74(2)	17.15^c^
O_2_	12	9.39	9.12(5)	9.69	9.59(4)	

a Number of qubits required for wave function mapping, and it corresponds to the number of spin orbitals in the active space.

b Ref. 81.

c Ref. 82.

Because the experimental *J* values are available for these systems, we have examined basis set dependence on the *J* values by using STO-3G and 6-311++G** basis sets. The theoretical and experimental *J* values of the triplet ground state species under study are summarised in Table 1. In Table 1, the *J* value obtained from the BxB algorithm is the average value of five numerical simulations. The standard deviation of the *J*(BxB) is less than 0.05 kcal mol^−1^. Again, the BxB algorithm reproduced the *J*(CAS-CI) value within 1 kcal mol^−1^ of errors. The deviation of the *J*(BxB) value from the *J*(CAS-CI) is somewhat large in NH and OH^+^ with 6-311++G** basis set. The Trotter decomposition is majorly responsible for this deviation. In fact, by doubling the Trotter slice *N* in eqn (12), the Δ*J*(BxB − full-CI) becomes −0.20 and −0.26 kcal mol^−1^ for NH and OH^+^, respectively.

As expected, the *J* value calculated by using 6-311++G** basis set is closer to the experimental *J*, compared with the minimal basis set STO-3G.

### 3.3 Triple bond dissociation in N_2_ molecule

Applicability of the BxB algorithm is not limited to the two-spin systems. As stated in theorem 2, the BxB algorithm enables us to infer the *J* value if the system can be approximated by the two-site Heisenberg spin Hamiltonian given in eqn (16) with effective spins *S̃* ≥ 1/2. Here, we demonstrate the *J* value calculation in the triple bond dissociation in N_2_ molecule with the (6e, 6o) active space consisting of valence orbitals participating in the triple bond (σ/σ* and π/π* orbitals).

By using the STO-3G basis set, |*Ψ*_BS_〉 converges to the open shell state with six unpaired electrons, those of two occupy the σ-type orbitals and the rest distribute to the π-type orbitals, at the bond distance *R*(N–N) = 2.1 Å and longer. |*Ψ*_BS_〉 is approximated by the linear combinations of four spin states as given in eqn (27).27
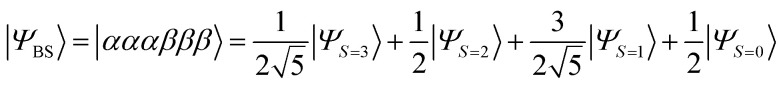


By assuming the Heisenberg spin Hamiltonian with effective spins *S̃* = 3/2, the spin-triplet (*S* = 1), spin-quintet (*S* = 2), and spin-septet (*S* = 3) states locate at 2*J*, 6*J* and 12*J*, respectively, above the spin-singlet ground state. In reality, the spin state energy gap is not exactly following this relationship because the exchange interaction between electrons in the σ-type orbitals is different from that in the π-type orbitals. The BxB algorithm calculates an effective *J*, rather than individual *J* values.

**Fig. 5:**
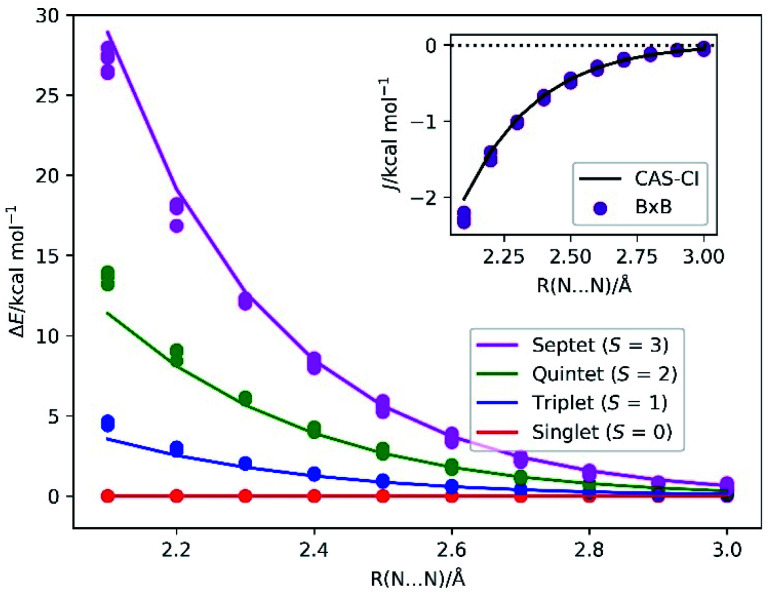
The energy diagram of the low-lying electronic states in the triple bond dissociation of N_2_. Solid lines specify the CAS-CI/STO-3G values, and circles denote the energy levels calculated from the *J*(BxB) obtained from the numerical quantum circuit simulations. Inset: the *J* value obtained from the CAS-CI/STO-3G calculations and from the BxB quantum algorithm simulations. The *J*(CAS-CI) is an average value of *J* computed from the relationship Δ*E*_S–T_ = 2*J*, Δ*E*_S–Q_ = 6*J* and Δ*E*_S−7_ = 12*J*.

The energy diagrams of the low-lying electronic states of N_2_ molecule at the bond length *R*(N–N) range from 2.1 to 3.0 Å are depicted in Fig. 5. The BxB algorithm predicted the trend of the spin state energy gap in a quantitative manner. Inset of Fig. 5 illustrates the *J* values obtained from the BxB algorithm and the CAS-CI/STO-3G calculation. The *J* value obtained from the BxB algorithm agree with the CAS-CI results within 0.2 kcal mol^−1^ of tolerance. The deviation from the *J*(CAS-CI) is smaller for the longer N–N distance, where the Heisenberg spin Hamiltonian with effective spins becomes a good approximation. Even for the systems with six unpaired electrons, Bayesian optimisation converged in about 10 iterations and the evolution time *t* is shorter than 300 atomic unit (see Fig. S3 in the ESI†).

## 4. Conclusions

In this work, we proposed a new quantum algorithm BxB for the calculation of the exchange coupling parameter *J* in Heisenberg spin Hamiltonian on quantum computers. The BxB algorithm enables us to calculate the *J* value within 1 kcal mol^−1^ of energy tolerance, regardless of the magnitude and absolute sign of the *J* value. The BxB algorithm has several advantages against the conventional *J* value calculations using QPE: the BxB algorithm directly calculates the spin state energy gap without inspecting the total energy of the individual spin states. The evolution time *t* to obtain the *J* value within 1 kcal mol^−1^ of tolerance is about 300 atomic unit in BxB, which is noticeably shorter than that in QPE (*t* ∼ 4000 a.u. or even longer). The conventional QPE requires good initial guess wave functions having sufficiently large overlap with the full-CI wave function because the energy of which electronic state is obtained is probabilistic. The BxB algorithm uses a broken-symmetry wave function consisting of one Slater determinant, and it is very easy to prepare on quantum computers. The QPE-based full-CI requires many controlled-*R*_*z*_ operations to calculate the energy, which should be executed on quantum computers with high fidelity. By contrast, no controlled-*R*_*z*_ operation is needed in the quantum simulation of the time evolution of wave functions in the BxB algorithm.

In this study, the numerical quantum circuit simulations were performed under the noise-free model, in which all quantum gates are executed perfectly and qubits possess infinitely long coherence time. In real quantum computing devices, noises and errors arising from inaccurate quantum gate operations and decoherences are inevitable. However, we expect that the BxB algorithm is more robust against these errors compared with the conventional QPE, because in the BxB algorithm the wave functions of high-spin and low-spin states are manipulated simultaneously by utilising the quantum superposition. In the BxB algorithm, randomly occurring errors and noises act similarly on the high-spin and low-spin states, and such errors can be cancelled out to some extent in the calculation of energy gaps. Also, the BxB algorithm is robust against measurement errors, because the BxB algorithm calculates a posterior distribution, rather than a point estimate. It should be also emphasised that the number of iterations required for achieving the convergence does not increase against the system size and the number of basis functions, because the BxB algorithm optimises a single parameter *j*. In addition, the BxB algorithm is free from the barren plateaus problem that is present in variational quantum eigensolver,^83^ because the evolution time length *t* is set to be as *P*(0) changes substantially in the range of *μ* − *σ*^2^ < *j* < *μ* + *σ*^2^.

The BxB algorithm needs (2*n* + 1) qubits to implement the computation for the system having the number of spin orbitals, *n*. This requirement hampers the BxB algorithm compared with the iterative QPE, which uses (*n* + 1) qubits. However, the (*n* + 1) qubits out of (2*n* + 1) can be initialised just before the SWAP test, and the number of qubits which are necessary to retain the quantum superposition during the time evolution simulation is the same for the iterative QPE and the BxB algorithm. Also, the ***S***^2^ operator only acts on the singly occupied molecular orbitals (SOMOs) and therefore it is possible to perform the SWAP test using qubits which store the occupation number of SOMOs in |*Ψ*_BS_〉 only. By adopting this approximation the number of qubits can be reduced to (*n* + 2 × *n*_spin_ + 1), where *n*_spin_ is the number of unpaired electrons in |*Ψ*_BS_〉. Using this strategy for the calculation of the *J* value in carbon atom with 6-311++G** basis set, we obtained *J*(BxB) = 18.17 ± 0.01 kcal mol^−1^, which is very close to that obtained by the SWAP test with all the qubits (*J* = 18.14 ± 0.01 kcal mol^−1^).

The BxB algorithm in the current form is applicable only for the systems whose electronic structure can be well approximated by the two-site Heisenberg spin Hamiltonian. However, this limitation originates from |*Ψ*_BS_〉. Importantly, theorem 1 is general and it always holds. This fact insists that we can calculate the energy gap between two states belonging to different spin multiplicities using the BxB-analogue quantum algorithms, if we can prepare the quantum state in superposition of high-spin and low-spin states. Combinations of the BxB algorithm and adiabatic state preparation (ASP) technique^23,84^ can potentially open the door to such applications. Another important extension of theory is applications to the molecules with three or more spin centres like triradicals and tetraradicals. The possibilities of these methodologies will be discussed in the forthcoming papers.

## Author contributions

K. Sugisaki, K. Sato, and T. Takui planned and conducted the project. K. Sugisaki proposed the theory and performed numerical simulations. All the other authors discussed the results. K. Sugisaki and T. Takui wrote the paper.

## Conflicts of interest

There are no conflicts to declare.

## Supplementary Material

SC-012-D0SC04847J-s001
