# Supplementary material for: A quantum algorithm for spin chemistry: a Bayesian exchange coupling parameter calculator with broken-symmetry wave functions
Source: Chem Sci. 2020 Dec 24;12(6):2121–32. doi: 10.1039/d0sc04847j (PMC8179312; doi:10.1039/d0sc04847j)
Supplement: SC-012-D0SC04847J-s001 [file SC-012-D0SC04847J-s001.pdf]

Electronic Supplementary Material (ESI) for Chemical Science.
This journal is © The Royal Society of Chemistry 2020

Table of Contents

		Page
**1.**	**Definition of quantum gates**	S2
**2.**	**Construction of quantum circuits of the time evolution operators**	S4
**3.**	**Computational conditions for the numerical simulation of the BxB algorithm**	S5
**4.**	**Convergence behaviour of the BxB quantum algorithm**	S7
**5.**	**References**	S8

1. Definitions of quantum gates

In quantum computer qubits can be in an arbitrary superposition of $|0\rangle$ and $|1\rangle$ states, as given in eqn (S1).

$$|\varphi\rangle = c_0|0\rangle + c_1|1\rangle \quad \text{(S1)}$$

Here, c_0 and c_1 are complex numbers satisfying a normalisation condition given in eqn (S2).

$$|c_0|^2 + |c_1|^2 = 1 \quad \text{(S2)}$$

The quantum state $|\varphi\rangle$ in eqn (S1) can be represented by a matrix as follows:

$$|\varphi\rangle = \begin{pmatrix} c_0 \\ c_1 \end{pmatrix} \quad \text{(S3)}$$

Quantum gates acting on one qubit can be expressed by a (2 × 2) unitary matrix and the quantum state after the quantum gate application can be calculated by matrix algebra. For example, the quantum state after the application of an Hadamard gate can be calculated as in eqn (S4).

$$H_d|\varphi\rangle = \frac{1}{\sqrt{2}}\begin{pmatrix} 1 & 1 \\ 1 & -1 \end{pmatrix}\begin{pmatrix} c_0 \\ c_1 \end{pmatrix} = \frac{1}{\sqrt{2}}\begin{pmatrix} c_0 + c_1 \\ c_0 - c_1 \end{pmatrix} \quad \text{(S4)}$$

The circuit symbols and matrix representations of quantum gates majorly used in quantum chemistry simulations are summarised in Table S1. In the quantum circuit, the horizontal lines specify a qubit or *n*-qubit quantum register, and squares, circles, and vertical lines represent quantum gates, which are applied to qubits from left to right order.

Table S1. Graph and matrix representations of quantum gates.

Gate	Circuit symbols	Matrix representations
Hadamard (H_d)	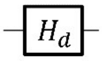	$\frac{1}{\sqrt{2}}\begin{pmatrix}1 & 1\\ 1 & -1\end{pmatrix}$
Pauli-X	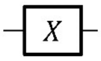	$\begin{pmatrix}0 & 1\\ 1 & 0\end{pmatrix}$
Pauli-Y	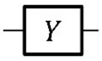	$\begin{pmatrix}0 & -i\\ i & 0\end{pmatrix}$
Pauli-Z	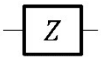	$\begin{pmatrix}1 & 0\\ 0 & -1\end{pmatrix}$
$R_x(\theta)$	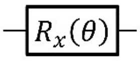	$\begin{pmatrix}\cos\frac{\theta}{2} & -i\sin\frac{\theta}{2}\\ -i\sin\frac{\theta}{2} & \cos\frac{\theta}{2}\end{pmatrix}$
$R_y(\theta)$	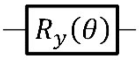	$\begin{pmatrix}\cos\frac{\theta}{2} & -\sin\frac{\theta}{2}\\ \sin\frac{\theta}{2} & \cos\frac{\theta}{2}\end{pmatrix}$
$R_z(\theta)$	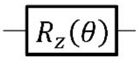	$\begin{pmatrix}e^{-i\theta/2} & 0\\ 0 & e^{i\theta/2}\end{pmatrix}$
$T_z(\theta)$	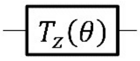	$\begin{pmatrix}1 & 0\\ 0 & e^{i\theta}\end{pmatrix}$
Controlled-NOT (CNOT)	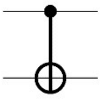	$\begin{pmatrix}1 & 0 & 0 & 0\\ 0 & 1 & 0 & 0\\ 0 & 0 & 0 & 1\\ 0 & 0 & 1 & 0\end{pmatrix}$
SWAP	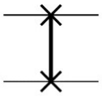	$\begin{pmatrix}1 & 0 & 0 & 0\\ 0 & 0 & 1 & 0\\ 0 & 1 & 0 & 0\\ 0 & 0 & 0 & 1\end{pmatrix}$
Toffoli (CCNOT)	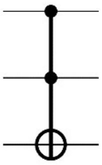	$\begin{pmatrix}1 & 0 & 0 & 0 & 0 & 0 & 0 & 0\\ 0 & 1 & 0 & 0 & 0 & 0 & 0 & 0\\ 0 & 0 & 1 & 0 & 0 & 0 & 0 & 0\\ 0 & 0 & 0 & 1 & 0 & 0 & 0 & 0\\ 0 & 0 & 0 & 0 & 1 & 0 & 0 & 0\\ 0 & 0 & 0 & 0 & 0 & 1 & 0 & 0\\ 0 & 0 & 0 & 0 & 0 & 0 & 0 & 1\\ 0 & 0 & 0 & 0 & 0 & 0 & 1 & 0\end{pmatrix}$

2. Construction of quantum circuits of the time evolution operators

The quantum circuit corresponding to a time evolution operator can be constructed by combining the quantum gates given in Table S1. The quantum circuit corresponding to the time evolution operator $exp(- iwX_1Z_2Z_3X_4t)$ is illustrated in Figure S1.

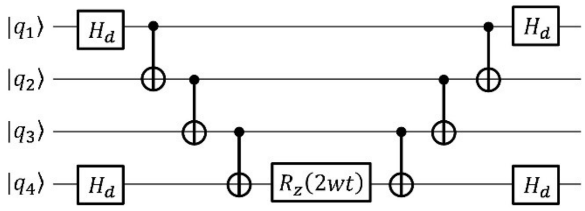

Figure S1. A quantum circuit for the time evolution operator $exp(- iwX_1Z_2Z_3X_4t)$.

It should be noted that the time evolution operators are applied conditional on the qubit for readout in the quantum phase estimation algorithm: Apply the time evolution operator if and only if the control qubit is in $|1\rangle$ state. Such operations can be done by substituting the $R_z(2wt)$ gate in Figure S1 by the controlled-$R_z(2wt)$ gate. As a result, the quantum circuit for QPE contains many controlled-R_z gates. Precise execution of the controlled-R_z gate is a key ingredient to accurately obtain the full-CI energy.

By contrast, in the BxB quantum algorithm, the time evolution operators are applied without any control qubit, which is easier to be implemented in quantum computing with high fidelity.

3. Computational conditions for the numerical simulations of the BxB algorithm

In order to demonstrate the BxB quantum algorithm we developed a numerical quantum circuit simulation program using Python with OpenFermion[S1] and Cirq[S2] libraries. The time evolution operator $U(j,t)$ can be decomposed by the product of the conventional time evolution operator $U(H,t) = exp(-iHt)$ and the time evolution operator of $j\mathbf{S}^2$ term $U(jS^2,t) = exp(-ijS^2t)$ as in eqn (S5) without loss of generality, because H and $\mathbf{S}^2$ commutes.

$$U(j,t) = \exp\{-i(H + jS^2)t\} = \exp(-iHt) \times \exp(-ijS^2t) = U(H,t) \times U(jS^2,t) \qquad \text{(S5)}$$

The time evolution operators $U(H,t)$ and $U(jS^2,t)$ are constructed by applying the JWT and generalised spin coordinate mapping (GSCM), respectively. The GSCM is proposed by us to efficiently simulate spin operations on quantum computers.[S3] Details of the GSCM can be found elsewhere.[S3,S4] We also reported that $U(jS^2,t)$ under the GSCM is robust to Trotter decomposition errors compared with that constructed by using JWT.[S4]

In the time evolution quantum simulation, we adopted the second-order Trotter decomposition given in eqn (S6) with $t/N = 1$. The same equation is given as eqn (12) in the main text.

$$U \approx \left[\prod_{m=1}^{M} exp(-iw_m P_m t/2N) \times \prod_{m=M}^{1} exp(-iw_m P_m t/2N)\right]^N \qquad \text{(S6)}$$

It is known that the Trotter decomposition error depends on the ordering of Pauli strings in eqn (S6).[S5,S6] In this work, we used a "magnitude ordering", where Pauli strings are ordered by the absolute value of the norm of Pauli strings $|w_i|$, for the Trotter decomposition of $U(H,t)$. The magnitude ordering is not suitable for the Trotter decomposition of $U(jS^2,t)$ because the norm of the terms in $\mathbf{S}^2$ operator is uniform. The $\mathbf{S}^2$ operator in the second quantised form is given in eq S7. We used a lexicological ordering in the Trotter decomposition to simulate $U(jS^2,t)$. Under the lexicological ordering, the GSCM gives noticeably smaller Trotter decomposition errors than the JWT in six molecular orbital systems (12 qubit simulations).[S4]

$$S^2 = \sum_{p,q}\left[\frac{1}{4}\left(a^\dagger_{p\alpha}a_{p\alpha}a^\dagger_{q\alpha}a_{q\alpha} + a^\dagger_{p\beta}a_{p\beta}a^\dagger_{q\beta}a_{q\beta} - a^\dagger_{p\alpha}a_{p\alpha}a^\dagger_{q\beta}a_{q\beta} - a^\dagger_{p\beta}a_{p\beta}a^\dagger_{q\alpha}a_{q\alpha}\right) + \frac{1}{2}\left(a^\dagger_{p\alpha}a_{p\beta}a^\dagger_{q\beta}a_{q\alpha} + a^\dagger_{p\beta}a_{p\alpha}a^\dagger_{q\alpha}a_{q\beta}\right)\right] \qquad \text{(S7)}$$

The BxB quantum algorithm uses $|\Psi_{BS}\rangle$ as an input wave function. However, in $|\Psi_{BS}\rangle$ the spatial part of the α- and β-spin orbitals are different, and spin operations associated with the $\mathbf{S}^2$ operator are non-trivial. We avoided this difficulty by exploiting the following procedures. At first, calculate $|\Psi_{UHF}\rangle$ using the conventional approach, and then natural orbitals are constructed by diagonalising the one-electron density matrix. The natural orbitals are eigenfunctions of the density operator with occupation numbers as their eigenvalues. The natural orbitals share the same spatial distributions between spin-α and β orbitals. After that, the localised molecular orbitals are constructed by mixing the singly occupied natural orbitals (SONOs)

having the unity occupation numbers. Then, one- and two-electron integrals h_{pq} and h_{pqrs} are computed using the localised orbital basis. These calculations were performed by using GAMESS-US program package.[S7]

In this work, we calculated the J value of H_2 molecule under covalent bond dissociation and those for C, O, Si, NH, OH^+, CH_2, NF and O_2, and N_2 molecule under triple bond dissociation. In the calculations of NH, OH, CH_2, NF and O_2 molecules, we used the geometrical parameters which have been experimentally determined: R(N–H) = 1.0362 Å in NH, R(O–H) = 1.029 Å in OH^+, R(C–H) = 1.085 Å and A(H–C–H) = 135.5° in CH_2, R(N–F) = 1.317 Å in NF, and R(O–O) = 1.208 Å in O_2. We have executed five numerical quantum circuit simulations for each molecular geometry.

4. Convergence behaviour of the BxB quantum algorithm

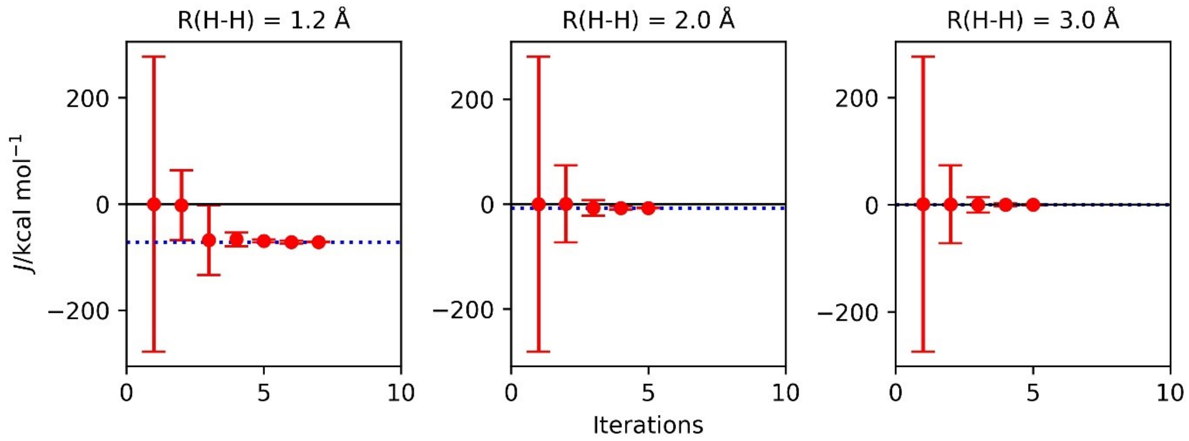

Figure S2. The posterior distributions obtained from the BxB algorithm in H_2 molecule at the atom–atom distance R(H–H) = 1.2, 2.0, and 3.0 Å. The dotted blue line specifies the *J*(full-CI/STO-3G) value. Red circles and error bars represent the mean and variance, respectively, of the posterior distribution.

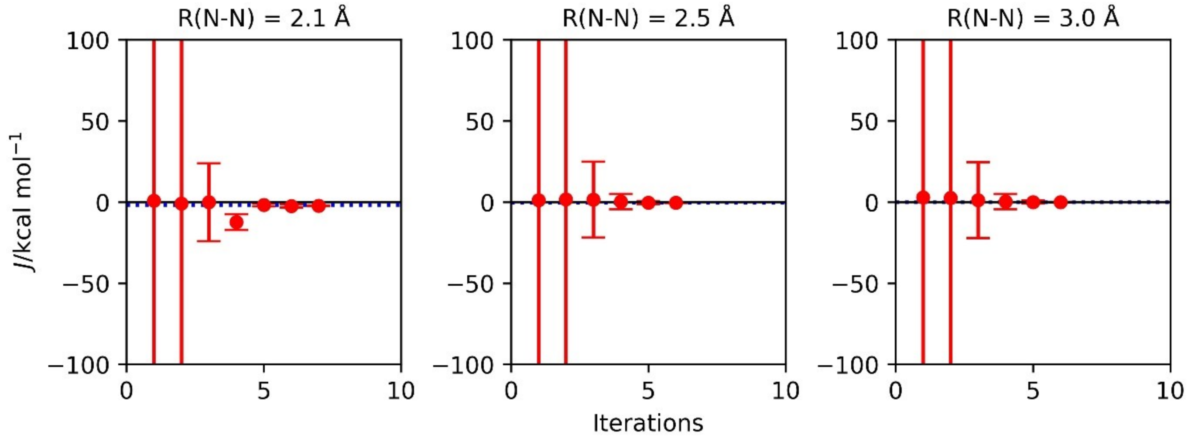

Figure S3 The posterior distributions obtained from the BxB algorithm in N_2 molecule at the atom–atom distance R(H–H) = 2.1, 2.5, and 3.0 Å. The dotted blue line specifies the *J*(CAS-CI(6e,6o)/STO-3G) value. Red circles and error bars represent the mean and variance, respectively, of the posterior distribution.

5. References

[S1] J. R. McClean, N. C. Rubin, K. J. Sung, I. D. Kivlichan, X. Bonet-Monroig, Y. Cao, C. Dai, F. E. Schuyler, C. Gidney, B. Gimby, P. Gokhale, T. Häner, T. Hardikar, V. Havlíček, O. Higgott, C. Huang, J. Izaac, Z. Jiang, X. Liu, S. McArdle, M. Neeley, T. O'Brien, B. O'Gorman, I. Ozfidan, M. D. Radin, J. Romero, N. P. D. Sawaya, B. Senjean, K. Setia, S. Sim, D. S. Steiger, M. Steudtner, Q. Sun, W. Sun, D. Wang, F. Zhang and R. Babbush, OpenFermion: the electronic structure package for quantum computers. *Quantum Sci. Tech.,* 2020, **5**, 034014.

[S2] Cirq. https://github.com/quantumlib/Cirq

[S3] K. Sugisaki, S. Nakazawa, K. Toyota, K. Sato, D. Shiomi and T. Takui, Quantum chemistry on quantum computers: quantum simulations of the time evolution of wave functions under the $\mathbf{S}^2$ operator and determination of the spin quantum number *S. Phys. Chem. Chem. Phys.,* 2019, **21**, 15356–15361.

[S4] K. Sugisaki, K. Toyota, K. Sato, D. Shiomi and T. Takui, A probabilistic spin annihilation method for quantum chemical calculations on quantum computers. *Phys. Chem. Chem. Phys.,* submitted.

[S5] A. Tranter, P. J. Love, F. Mintert and P. V. Coveney, A comparison of the Bravyi–Kitaev and Jordan–Wigner transformations for the quantum simulation of quantum chemistry. *J. Chem. Theory Comput.,* 2018, **14**, 5617–5630.

[S6] A. Tranter, P. J. Love, F. Mintert, N. Wiebe and P. V. Coveney, Ordering of Trotterization: impact on errors in quantum simulation of electronic structure. *Entropy,* 2019, **21**, 1218.

[S7] M. W. Schmidt, K. K. Baldridge, J. A. Boatz, S. T. Elbert, M. S. Gordon, J. H. Jensen, S. Koseki, N. Matsunaga, K. A. Nguyen, S. J. Su, T. L. Windus, M. Dupuis and J. A. Montgomery, General atomic and molecular electronic structure system. *J. Comp. Chem.,* 1993, **14**, 1347–1363.
